# Changes of Water Hydrogen Bond Network with Different Externalities

**DOI:** 10.3390/ijms16048454

**Published:** 2015-04-15

**Authors:** Lin Zhao, Kai Ma, Zi Yang

**Affiliations:** 1 School of Environmental Science and Engineering, Tianjin University, No. 92 Weijin Road, Tianjin 300072, China; E-Mail: makai@tju.edu.cn; 2 School of Chemical Engineering and Technology, Tianjin University, No. 92 Weijin Road, Tianjin 300072, China; E-Mail: yangzi83412832@163.com

**Keywords:** expanded structure, collapsed structure, hydrogen bonds, long-range effect, molecular dynamics, water cluster

## Abstract

It is crucial to uncover the mystery of water cluster and structural motif to have an insight into the abundant anomalies bound to water. In this context, the analysis of influence factors is an alternative way to shed light on the nature of water clusters. Water structure has been tentatively explained within different frameworks of structural models. Based on comprehensive analysis and summary of the studies on the response of water to four externalities (*i.e.*, temperature, pressure, solutes and external fields), the changing trends of water structure and a deduced intrinsic structural motif are put forward in this work. The variations in physicochemical and biological effects of water induced by each externality are also discussed to emphasize the role of water in our daily life. On this basis, the underlying problems that need to be further studied are formulated by pointing out the limitations attached to current study techniques and to outline prominent studies that have come up recently.

## 1. Introduction

Water, one of the most abundant molecular compounds existing in our planet, has a variety of functions as to its usage in production, biological reaction and so on, as a result of which, water is of great importance to our daily life. Ubiquity and anomalous properties such as phase anomalies, thermodynamic anomalies, are the main cause for arising broad interests in proceeding a vast amount of studies on water. Up to now, it is widely accepted that the anomalous nature of water depends on the localized and structured clustering and corresponding dynamics embedded in the infinite hydrogen bond (HB) network [1]. Nevertheless, resorting to different experimental and theoretical calculation techniques, e.g., infrared spectroscopy, Raman spectroscopy, molecular dynamic (MD) simulations, X-ray spectroscopy, we may obtain different or completely opposite interpretation of water HB network and corresponding dynamics.

Concerning the interpretation of HB network, the water structural model could be basically divided into two opposite categories, namely continuum model proposed by Röntgen [2], and mixture model presented by Bernal and Fowler [3], dating back to 1933 and 1892, respectively. The continuum model describes the HB network as fully hydrogen bonded (tetrahedrally coordinated) motif without any breakage in any case, but the O–H•••O angles and distances could be distorted under some circumstances [2], e.g., increasing temperature, elevating pressure. Many researchers have made a considerable amount of further studies on the basis of this model. Satisfyingly, the continuum model exerted a great power in explaining some anomalous behaviors of bulk-phase water [4]. The mixture model considers the bulk phase as an ensemble consisting of two basic components, an ice-like fraction with low density and a bond-free like fraction with high density [3], and the changes in their relative proportions would determine the physical properties of the ensemble. Previously, promising studies on HB dynamics and lone-pair electrons residing in a water molecule, employing an ultrafast laser beam [5,6] and X-ray absorption spectroscopy and X-ray emission spectroscopy (XAS/XES) [7,8,9], respectively, have demonstrated the existence of spectrally distinguishable HB configurations again, which is further confirmed by conducting MD simulations [10]. Very recently, some research focusing on the isosbestic point emerging in temperature-dependent vibrational spectra, brought the inconformity between the two models into our view again, as both could present plausible explanations on this spectrum feature [11,12,13,14]. So, one may get some clues to HB network by analyzing the changes in the properties of water induced by external factors, e.g., temperature, electromagnetic field.

Given a mutual interaction system comprising water and surroundings, the properties of water could reflect the influences of well-defined circumstance. For example, the refractive index would remarkably increase with the presence of magnetic field [15]. Moreover, according to opposite effects of ions on the properties of water, some researchers classified the ions into two categories, “kosmotrope” (order-maker) and “chaotrope” (disorder-maker) [16], which will be discussed in the following part. Meantime, we should keep in mind that the microscopic structure of water is closely related to their macroscopic properties and functions. Breaking hydrogen bonds of bulk water by raising temperature [17] could, consequentially, reduce the dynamic viscosity [18] and, conversely, monotonously increase the thermal expansivity [19]. Moreover, the changes in structure and function of biomacromolecule, and the formation of scale inside pipe or container, have direct relationship with the microscopic structure of water as well. So, water should never be considered simply as an inert solvent, on the contrary, it serves “magic” roles for its diverse usages and functions.

In this review we would like to pay much attention to relevant research progress in water structure and their variation behavior with surroundings. This interest is a consequence of the fact that the investigations on water structure are the basis with which we may bridge the gap between externalities and corresponding changes of water properties. Thereby, we could ask which factors may have an influence on water cluster, whether we can regulate some biological and physical processes by controlling the microstructure of water, which acts as solvent or constituent of specific molecule. We, however, apologize beforehand for any important studies or contributions that may have been inadvertently omitted.

The framework of this review is arranged as follows: First, discussion of the variation of structure and dynamics of water HB network with temperature; second, tentatively deducing the structural motif of water cluster by further incorporating pressure-related research outcomes; third, elaborating on the role of solutes in determining the structure and dynamics of HB; fourth, proposing puzzling effect of three external fields on water cluster; and finally, outlining a summary and outlook in view of the progress and promise of relevant fields.

## 2. Water Structure Changes with Temperature

It is widely known that water could exist in solid, liquid and gas phase, which is greatly dependent on its temperature, although it is also affected by ambient pressure. The structure, vibrational-rotational dynamics, and correspondingly the functions are completely different from each other, even when made of the same kind of molecule. In this Section, we discuss the characters of four species of water, namely supercooled water, ambient water, supercritical water (SCW) and gaseous water, which are sensitive to surrounding temperature.

### 2.1. Supercooled Water

If enough care is taken to reduce the directional tendency of water HB to avoid heterogeneous nucleation of liquid water by eliminating the impurities and cooling at fast speed and proper pressure, supercooled water could be obtained below the melting temperature.

**Figure 1:**
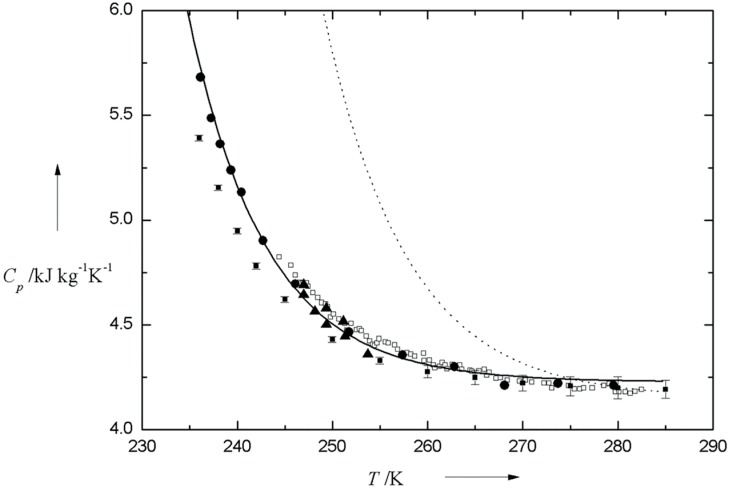
Variation of *C*_p_ with temperature. Specifically, solid triangle, solid circle, solid square with error bar, hollow square are denoted as the value of *C*_p_ determined by Bertolini *et al.* [28], Angell *et al.* [20,21], Archer and Carter [29], and Tombari *et al.* [30], respectively. For better comparison, the solid line and dashed line, representing the simulation results from IAPWS-95 and IAPWS-84, respectively [31], are also shown.

**Figure 2:**
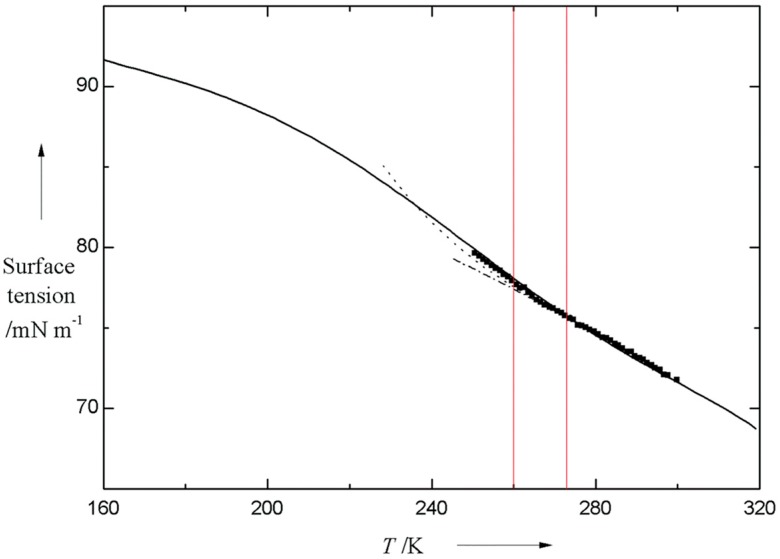
Comparison of surface tension as a function of temperature. Dot dashed line: calculated from early IAPWS equation [32], dashed line: calculated from advanced IAPWS equation by incorporating a new term [33], solid line: calculated by employing a two-state model [27], solid square: experimental results by Hacker [26]. The two red straight lines are schematic symbols to guide the eyes to the inflection point existing between the lines. It should be noted that there is a big discrepancy in surface tension between two-state model and developed IAPWS equation below the temperature of inflection point, even though a remarkable agreement existing at the higher temperatures.

Because of the slow dynamics of HB network, there have been a vast amount of experimental and theoretical studies on characterizing the properties of supercooled water. The variation of isobaric heat capacity (*C*_p_) with temperature measured by different researchers could be seen from Figure 1. From this figure, we can see that the ascending tendency of *C*_p_ would persist within a large supercooling range, even down to 236 K [20,21]. Given that the macroscopic parameters could reflect the microscopic structural features, the change in *C*_p_ is an indication of strengthening of hydrogen bonding with temperature descending. Moreover, surface tension (γ) or viscosity (η) is a good marker for changes in the geometry and strength of HB network, which has been confirmed by us [22,23] and other researchers [18,24,25], aided by high resolution γ, η test instruments. The changing tendency of surface tension with temperature could be seen from Figure 2. Floriano and Angell [24], Hacker [26], and Trinh and Ohsaka [25], all unexceptionally found the increase of surface tension of supercooled water with temperature descending, which is due mainly to the strengthening of HB network (see Figure 2). Additionally, Floriano and Angell [24], and Hacker [26], also found an inflection point existing around 268 K, which was further confirmed by employing a two-state thermodynamic model [27].

Besides normal supercooled water, another magic state, namely deeply supercooled water, also exists for water within specific temperature and pressure ranges, and is normally distinguished by the transition temperature of *T*_X_ and *T*_g_ denoting crystallization and glass transition temperature, respectively [34,35]. As temperature descends beyond *T*_X_, crystalline ice would transform into deeply supercooled water, which would further transform into amorphous ice with temperature decreasing below *T*_g_. Compared with crystalline ice, all the three phases have more randomized HB network, and hence, one may find some clues to water cluster by conducting analogy analysis on inheritance relationship among the three distinct phases. Poole *et al.* [36], associating the anomalous behavior of supercooled water with the transition between low density amorphous ice (LDA) and high density amorphous ice (HDA), formulated the idea that supercooled water was continuously connected to LDA ice. In other words, supercooled water is composed mainly of LDA like structure motif. However, supercooled water itself also experiences structure changes reflected by the macroscopic parameters mentioned above [21,24]. Consequently, one can make a presumption that, resemble to amorphous ice, supercooled water consists of two components as well, low density liquid (LDL) and high density liquid (HDL), as indicated by Mishima and Stanley [34].

In nowadays, the two-state model has been widely accepted and employed to explain the experimental results of supercooled water, such as the inflection point of surface tension as a function of temperature [27]. Moreover, the supercooled water is characterized by more ordered structure relative to ambient water, and the two structural motifs have been deduced from the variation of radial distribution function by using X-ray diffraction spectroscopy [37]. So it seems feasible to employ the two-state model to explain the variation of thermodynamic properties as a function of temperature. In effect, in 2000, Chaplin has proposed a two-state water network fluctuating between expanded structure (ES) and collapsed structure (CS), formed by identical units of 14 water molecules, to account for many of the anomalous properties of water [38]. Here and in the following Sections, we will manifest the relationship of ES-CS model with other water models and tentatively give explanations on water-mediated physicochemical and biological processes by applying these water models.

With temperature decreasing, supercooled water is prone to form ordered water structure with entropy decreasing. So from the viewpoint of thermodynamics, supercooled water is characterized by very high heat capacity as shown in Figure 1. Considering the good liquidity, this material and its upgraded version, ice slurry, have broad application space in the field of heating ventilation air conditioning and frozen storage.

### 2.2. Ambient Water

Ambient water, existing within a large temperature span from 0 to 100 °C, is the most ubiquitous and commonly visible state of water in our daily life. For this reason, with the help of theoretical calculations and modern experimental instruments, structure and dynamics of ambient water have been the focus of considerable studies.

Ionization, represented by *K*_w_, is associated strongly with macroscopic parameters, e.g., pH, electric conductivity, and should not be considered simply as the presentation of hydrogen ion (H^+^) and hydroxyl ion (OH^−^), but a dynamic process controlled by the generation, separation and recombination of the ion pairs. The separation process, constrained by the connectivity of HB network, could be depicted by the Grotthuss mechanism [39], whereby hydrogen transfers from one water molecule to the next. In effect, *K*_w_ is strongly temperature dependent, and specifically, increases from 0.001 × 10^−14^ mol^2^·L^−2^ at −35 °C, to 0.991 × 10^−14^ mol^2^·L^−2^ at 25 °C, to 9.311 × 10^−14^ mol^2^·L^−2^ at 60 °C, which corresponds to pH of 8.5, 7.0 and 6.5, respectively. So the inherent connectivity of water structure would change with temperature as well.

Non-overlapping configuration of positive and negative charge center of an isolated water monomer is responsible for the comparatively large dipole moment of 1.855 D and anomalous behaviors of bulk water. According to Gregory *et al.* [40], the dipole moment of small water cluster, *n* = 1–8, climbs up to ~2.76 D from 1.865 D as *n* increases. Further, this value in condensed phase increases up to 2.4–2.6 D, which is attributable to the polarization of the ensemble [41]. So in the presence of HBs, the center of positive and negative charge departs from each other even further with the cluster growing up, consequentially, the average dipole moment appears to increase upon cooling. However, dipole moment is a microscopic parameter, which could not be detected directly up to now. On the basis of the Onsager theory, Equation (1) is deduced to bridge the gap between dipole moment (μ) and measurable parameter, dielectric constant (ε).

(1) $$\frac{\left(\varepsilon_{0}-\varepsilon_{\infty })(2\varepsilon_{0}-\varepsilon_{\infty }\right)}{\varepsilon_{0}{\left(\varepsilon_{\infty }+2\right)}^{2}}=\frac{4\pi N_{\mathrm{AV}}g\eta^{v^{2}}}{9kTV_{\varepsilon }}$$

where ε_0_, ε_∞_, *g*, $\mu^{v^{2}}$, *k*, *T*, *N*_AV_ and *V* are measured dielectric constant, the dielectric constant at a frequency sufficiently low for atomic and electronic polarization but sufficiently high for intermolecular relaxation processes, parameter of local ordering degree, dipole moment in vapor phase, Boltzmann constant, thermodynamic temperature in K, Avagadro number and molar volume, respectively.

In this context, Suresh and Naik [42] related the parameter *g* to another parameter *P*, probability of central water molecule hydrogen bonding to surrounding molecules at an instant. Even though the cooperativity of water HB is disappointedly unconsidered within the theory, the good agreement of the fitting curve with experimental data has proved the variation of dielectric constant is determined by dipole moment and extent of random orientation of water molecule, which is influenced by the ambient temperature. Further, given the almost identical importance of average dipole moment and local alignment of dipoles in determining the unusually large dielectric constant of liquid water [43], one can conclude that temperature elevation would lead to the decrease of dipole moment and random orientation of water dipoles by breaking HBs simultaneously.

Water molecule involves different kinds of motions simultaneously, vibration, rotation and transition, which enables the appearance of a complex absorption profile affected by surrounding water molecules within several frequency ranges. The wavenumber ranging from 3000 to 3700 cm^−1^, 1000 to 2000 cm^−1^, and 0 to 200 cm^−1^, have been experimentally shown to have evident absorption ascribed to stretching vibration, bending vibration and rotation of H_2_O, respectively. Consequently, a vast amount of studies focusing on the changes in spectrum with temperature were conducted and found opposite variation behaviors of stretching and bending vibrational peak by employing IR, Raman spectroscopy [14,44,45,46], which can be attributed to the enhanced thermal fluctuations and weakened limitations on stretching vibration caused by the decreased importance of intermolecular hydrogen bonding at higher temperature. The temperature effect on HB strength could also be obtained from soft X-ray Raman spectroscopy (XRS) and XAS by probing excitations of oxygen 1*s* electrons to the valence unoccupied molecular orbitals of water [47,48].

With temperature increasing, the stretching vibrational peak not only shows the broadening and blue-shifting behavior, but also gives rise to a remarkable intersection point, isosbestic point, which has been studied to illustrate microstructure of water HB network in recent years. The term “isosbestic point” refers to the temperature-independent frequency at which a series of spectral lines cross. The fixed isosbestic point is often regarded as the result of equilibrium between hydrogen-bonded (HB) and non-hydrogen-bonded (NHB) OH-stretching component [14,49], and so as an indicator of the mixture model. From the viewpoint of thermodynamics, an excellent agreement of experimental Δ*E* obtained by conducting a van’t Hoff plot using the IR absorption intensities of two populations, with other theoretical values [50], further confirmed the two-component theory for liquid water. Meanwhile, a Raman study decomposing absorption band into five subclasses, proved the existence of multi-component in liquid water in view of the remarkable goodness of fit to van’t Hoff equation [13]. In addition, by decomposing the dielectric constants into four component modes, a THz TD-ATR study found the fast relaxation mode was the collisional relaxation process originating from isolated water molecules, which implied ambient water should be regarded as two component mixtures [51]. Interestingly, another dielectric study also showed a magic point, isopermitive point, and gave an explanation on this point within the framework of two-component model [52]. However, the isosbestic point can also be interpreted from the viewpoint of tetrahedral arrangement of water molecules, or in other words continuum model [12], on condition that the variations in temperature and pressure are less than 100 K and 10^4^ atm, respectively [11], which is the prerequisite for the insensitivity of equilibrium distribution of relevant coordinates to the surroundings. Consequently up to now, we still cannot declare which is the suitable model by merely eyeing on the two magic points. The interpretation of the two points and corresponding conditions are presented in Table 1 for comparison.

**Table 1:** Interpretation of isosbestic and isopermitive point for liquid water.

Kind of Point	Method	Interpretation of Point	Detailed Classification	Temperature Ranges (°C)	References
isosbestic point	near-IR	MCM ^a^	HB-NHB ^b^	5–25	Worley and Klotz [49]
isosbestic point	Raman spectra	MCM ^a^	HB-NHB ^b^	3–85	Walrafen *et al.* [14]
isosbestic point	Raman spectra	MCM ^a^	free OH, DA, DDA, DAA, DDAA	−25–25	Sun [13]
isosbestic point	Raman spectra and thermodynamic calculation	MCM ^a^	CW-UCW ^c^	25–60	Nino *et al.* [50]
isosbestic point	molecular simulations	CM ^d^	none ^e^	0–100	Geissler [11]
isosbestic point	Raman spectra and MC simulation	CM ^d^	none ^e^	0–100	Smith *et al.* [12]
isopermitive point	Dielectric spectra	MCM ^a^	ions and dipoles	26–40	Angulo and Mercado [52]

^a^ MCM, multi-component model; ^b^ HB and NHB, hydrogen bonded and non-hydrogen bonded, respectively; ^c^ CW and UCW, correlated and uncorrelated water, respectively; ^d^ CM, continuous model; ^e^ The network is continuous and so could not be further classified into subcategories.

**Figure 3:**
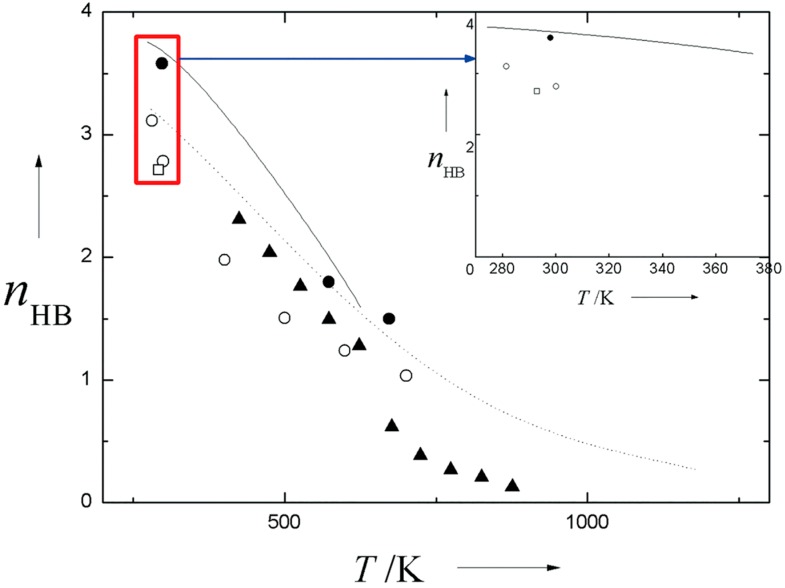
Comparison of $n_{\mathrm{HB}}$ obtained from various molecular simulations and spectrum studies. Dot line: MC (TIP4P) simulations at 100 MPa [56], solid line: molecular simulations based on cluster theory [42], solid triangle: estimations from NMR shift at pressure ranging from 2.5 to 40 MPa [53], solid circle: estimations from neutron diffraction at 0.1, 9.5, 80 MPa, respectively [54], hollow circle: MD calculations based on fluctuating-charge TIP4P-FQ model [55], hollow square: estimations from Raman spectrum at ambient condition [13]. Four estimated $n_{\mathrm{HB}}$ and one line are shown in the inset to give a better comparison for ambient water at 0.1 MPa.

The changes in individual water molecule could also be reflected by the average hydrogen bonding number ($n_{\mathrm{HB}}$). Hoffmann and Conradi [53], performed a proton NMR study on water with large temperature span ranging from 150 to 600 °C. By assuming a linear relationship between the chemical shifts and the degree of hydrogen bonding, they concluded that the weakening of hydrogen bond and the continuous decrease of $n_{\mathrm{HB}}$ occurred simultaneously with temperature up to the critical point. Moreover, the changing tendency of $n_{\mathrm{HB}}$ could also be manifested from simulation studies, e.g., calculated neutron diffraction spectrum based on the empirical potential Monte Carlo (EPMC) method [54], calculated dielectric constant within the framework of statistical mechanical principles [42], calculated dielectric constant using fluctuating-charge TIP4P-FQ model [55]. Recently, the Raman spectrum was shown to have the potential in obtaining $n_{\mathrm{HB}}$, *i.e.*, 2.71 at the condition of 293 K and 0.1 MPa [13]. From Figure 3, one could find the differences and changing tendency of $n_{\mathrm{HB}}$ estimated from molecular simulations and spectrum studies. Keep in mind that when performing molecular simulations, the energetic or geometry criterion for hydrogen bonding is the prerequisite for defining $n_{\mathrm{HB}}$.

Diffraction method is excellent in visualizing the time-averaged microscopic geometries of HB network by providing the information on distances between the atoms of water molecules. This method is often coupled with theoretical calculations to test the correctness of a model. As temperature could induce the changes in HB configuration reflected by the parameters mentioned above, it is supposed that temperature variation may influence the O–O, O–H and H–H distances reflected by pair correlation function (PCF), *g*_OO_(*r*), *g*_OH_(*r*) and *g*_HH_(*r*), respectively. Recently, an MD simulations using high-LSI and low-LSI order parameters, gave remarkably accurate predictions for most PCF features obtained from wide-angle X-ray diffraction experiments, and further supported the coexistence of two different local structure, HDL- and LDL-like structure [57], similar to CS- and ES-like motif, respectively. Time-resolved infrared spectroscopy has the remarkable potential to provide unprecedented information on structure, especially for HB rearrangement dynamics in liquids. Given the exquisite sensitivity of the strength and lifetime of HB to temperature [58], the decay time constant extracted from pump-probe spectrum, manifests bimodal distribution in liquid water with the presence of two significantly different time scales. Further, the newly coming two dimensional infrared (2D IR) vibrational spectroscopy also indicates the existence of two distinct reorientational relaxation processes [59]. Thus, two structural motifs coexisting in liquid water are convincingly evidenced by long-range structural features and HB rearrangement dynamics.

Appealing to us, temperature-induced structure changes could further lead to water-mediated physical process alteration. Heating ambient water could be further used to proceed fast-freezing as indicated by Mpemba. Counter-intuitively, hot water (e.g., 85 °C) freezes faster than the same amount of cold water (e.g., 15 °C) under otherwise equal conditions. According to the preceding discussions, the liquid water with low temperature is prone to involve high concentration of ES clusters, e.g., icosahedral clusters, the liquid water with high temperature contains high concentration of less ordered CS clusters. Nevertheless, as for Ih, the hexagonal ice crystal is the major structural unit. So given that the clustering process from low-density water to hexagonal ice crystal would take some time [60], it seems to be reasonable that hot water with randomized CS cluster possesses a faster cooling rate. Another study by Ehre *et al.* [61], reported that the negatively charged material could lower down the freezing point to a deeper level. Therefore, one could speculate the faster cooling of hot water mainly results from the loss of the negatively charged nanobubbles upon heating. Even still controversial in exact mechanism, the Mpemba effect has potential to be used to get liquid water frozen quickly.

Water in our body bears multiple roles, e.g., reaction medium, buffer agent. Here, we focus on the water-mediated protein denaturation imposed by temperature variation. Incorporating non-polar groups of protein into solution can only lead to a limited increase in entropy of the system because of the incompatibility of this surface with surrounding water structure [62], therefore these groups are mostly buried inside of 3D protein to minimize such contact [63]. Given that the exposure of non-polar groups can encourage the formation of ES cluster at low temperature [64], the transfer of non-polar groups from interior to the bulk water would be the enthalpy favored process, which is responsible for the cooling denaturation. Just on the contrary, the breakage of hydrogen bond network caused by temperature elevation would be compensated by the entropic favored distortion of the system. As a result, the relatively constant body temperature of warm-blooded animal is responsible for the naturally functioning of proteins and other bio-molecules.

### 2.3. Gaseous Water

It is ubiquitous that gaseous water could coexist with liquid water within a wide range of temperature, even below its condensation temperature (*i.e.*, dew point) in the absence of nucleation-assisting substances. Based on the variational transition state theory (VTST) focusing on the dynamical quantities of clusters [65], it is believed that there is an equilibrium between evaporation and coagulation for gaseous and bulk water. So, identifying structural features of gaseous water is meaningful to fully understanding water structure.

Water vapor, a dispersed and discontinuous state, would involve more small water clusters relative to the bulk phase due mainly to weak HB and less constraint by surrounding water molecules. According to a theoretical study describing the nucleation kinetics [65], it is supposed to underscore the importance of water monomers in water vapor. As shown by experiments, water dimer does exist in artificial segmented phases, e.g., supersonic molecular beams [66], helium nanodroplets [67]. Very recently, the existence of water dimer in atmospheric condition was further experimentally confirmed by using a resonator spectrometer operating in the millimeter-wave band [68]. Given the more cooperativity and stronger hydrogen bonding, large cluster is prone to exist in ambient water characterized by large hydrogen bonding number ($n_{\mathrm{HB}}$ > 2.5, see the inset in Figure 3). By contrast, $n_{\mathrm{HB}}$ of water vapor would be smaller than 1 or even close to 0, which depends on a specific condition. As a whole, the size distribution of different water clusters at equilibrium would be characterized by higher mole fraction of monomer and dimer and only limited amounts of larger water clusters (*n* > 2) in water vapor. Moreover, even forming in a flash, the latter would break down into monomers and small clusters as temperature increasing [69].

The ideal structural model is one that could represent the properties of different forms of water. However, the original ES-CS model is not suitable for gaseous water anymore because of its intrinsic larger structural unit. Thus, a compromised way to extending the applicability of ES-CS model is to adjust the fixed structural unit into size-variable unit, namely, these identical structural units can not only spontaneously assemble into fully hydrogen bonded cluster, but can also be reshaped by variable surroundings. To further prove the suitability of this modified model, it is imperative to compare experimental properties with simulations results of water vapor in the future.

Fewer HBs and more interstitial spaces for water vapor than that for liquid water give rise to the sharp contrast in volume between the two phases, specifically, the volume of water vapor is 1603.6 times that of the same mass of liquid water at the boiling point and atmospheric pressure, and according to state equation, the volume of water vapor would increase further with temperature. Therefore, this anomalous behavior has been widely used in the steam engine and steam generator in the early 18th century. Naturally, gaseous water, as one constituent in atmosphere, is of paramount importance as for influencing the atmospheric chemistry, climate and homogeneous condensation, all of which may be related to the binary complex formed by water molecule and various atmospheric molecules, e.g., H_2_SO_4_, O_3_, N_2_ [70], through strong interactions (*i.e.*, dipolar interactions and hydrogen bonding). The strong interactions can alter electronic states of the monomeric chromophore [71], and further decrease transition energy barrier to facilitate the chemical reactions [72]. Besides the energetic point of view, a new research manifested the efficiency of evaluating the relative competing rates between reaction and dissipation in elucidating the role of water in chemistry [73]. Interestingly, water molecules in atmosphere can also play a role as inhibitor and anti-catalytic agent in the thermal and photochemical reactions when reagents hydrogen bonding to water molecule [72]. As a result, it is of great necessity to identify the cluster configuration and relaxation dynamics to assert the role of water molecule in affecting atmospheric chemistry aided by temperature- and humidity-dependent spectra.

### 2.4. Sub- and Supercritical Water

It is widely accepted that *T* = 647 K and *P* = 22.1 MPa is the key point for water, called critical point, whereby water would appear as a magic state with strong temperature- and pressure-dependence. Here, temperature-induced changes in parameters and spectrums are analyzed to illustrate the structural features of SCW and their applications in extraction and chemical reaction fields.

Similar to gaseous water, SCW as well involves distorted HB configurations reflected by the special parameters and spectrums. In 1993, Postorino *et al.* [74] interpreted neutron diffraction pattern of SCW as an indication of thoroughly absence of HB at subcritical temperatures. However, incorporating the inelasticity correction, one could apparently diminish the big discrepancy in site-site correlation functions from different experiments [54]. In this context, Kalinichev [75] further showed the independent sources of experimental and simulation results that all agreed with the existence of a certain degree of hydrogen bonding in SCW. One more step, by sophisticatedly interpreting X-ray Raman scattering spectrum, SCW can be viewed as a mixture composed of hydrogen bonded molecules and non-hydrogen bonded molecules [76], *i.e.*, inhomogeneous ensemble. So there is no doubt that HB is still present at supercritical conditions, but to what extent the water molecules hydrogen bonding to each other and the microscopic motif of HB network, necessitate further elaborations as follows.

As early as 1969, it was found that the dynamic viscosity of water manifested a downtrend with temperature varying from 100 to 1500 °C [77]. While, self-diffusion coefficient, being approximately proportional to the reciprocal of viscosity as illustrated by Stokes-Einstein equation, has been shown to ascend up to 68 × 10^−5^ from 45 × 10^−5^ cm^2^·s^−1^ with temperature ranging from 630 to 772 K [78]. Additionally, given that the dipole moment can directly reflect hydrogen bonding strength, its decrease from 2.60 D at ambient condition to 1.87 D at supercritical condition and the leveling up of randomized orientation of water molecules [55], can be ascribed to the weakening of hydrogen bonding with temperature. Analogous to the phase transition from ice to liquid water, the decrease of dipole moment, together with the variation behavior of viscosity and self-diffusion coefficient, are the consequence of less pronounced induction effect and disruption of cooperative HB network with temperature elevating, which makes sub- and supercritical water more resemble non-polar compound.

Focusing on the individual molecule and its first coordination shell, we would find the water molecules lose the feature of tetrahedral coordination and are characterized by smaller $n_{\mathrm{HB}}$ of 2.4 [75], which could be interpreted as the transformation of local configuration within the first neighboring shell from 3D tetrahedral structure to sheetlike trigonal structure [79]. There are also some hints from far- and mid-infrared spectroscopy, that evidence the weakening or even breakdown of water HB network as temperature ranging from ambient to supercritical conditions [80]. The relevant studies indicating the structural features of SCW are summarized in Table 2.

In summary, even if the energy of HB is still markedly larger than thermal energy at the critical temperature [81], compared to ambient water, SCW has lost extensive HB network in the extreme state as evidenced by the increased free volume of the molecules and average volume of the vacancies with temperature [82]. Correspondingly, SCW is broken into discontinuous fragments, *i.e.*, dimers and small clusters [83]. Because of the high difficulty in conducting measurements under supercritical conditions, the exact structural motif for SCW and corresponding evolutionary process with temperature are still far from being completely acquainted, which necessitates the researches on the long-range order and rearrangement dynamics of HB. Nevertheless, there are more HBs in SCW than that in water vapor, so the ES-CS model is very promising to be used in this phase to explain some anomalous behaviors of SCW that will be discussed in Section 3.2.

**Table 2:** Property changes and the corresponding presumption on structural features of SCW.

Parameters	Changing Trend with Temperature	Structural Features	References
dielectric constant	decreasing	Dipole moment decreasing and randomized orientation	Yoshii *et al.* [55]
electric conductivity	increasing	Transforming to ionic state	Chau *et al.* [84]
viscosity	decreasing	HB weakening ^a^	Lamb and Jonas [85]
self-diffusion coefficient	increasing	HB weakening ^b^	Lamb and Jonas [85] Brodholt and Wood [78]
HB vibration absorption	vanishment	HB breakage	Kalinichev [86]
OH stretching absorption	blue shift	HB breakage	Kohl *et al.* [80]

^a^ The authors observed the HB weakening by relating the spin-rotation relaxation time from NMR to viscosity from empirical calculations [77,87]; ^b^ Two studies independently got the same results by using NMR and MD simulations, respectively.

At sub- and supercritical conditions, water and non-ionic species, e.g., O_2_, N_2_, and even for organic compounds, are completely miscible. The abnormal solubility of organic compounds must be related to the loss of HB in SCW. Considering the unfavorable breakage of HB network to effectively incorporate organic molecules [88], the less dense regions which is one component of water medium, could serve as the ideal area for the solvation of organic molecules because of needlessness of unfavorable breakage of HB [76]. So, SCW, capable of dissolving O_2_ and most organics substances simultaneously, has been widely used as a good medium for increasing the probability of contact between two reactants with no interface transport limitations, which is the root of supercritical water oxidation (SCWO).

## 3. Pressure-Induced Changes of Water

From phase diagram with broad temperature and pressure ranges, one could see that some phase transition lines form small angle or even parallel to temperature axis, put another way, the corresponding phase transitions between different forms of ice are strongly pressure-dependent, which are essentially caused by pressure-induced micro-structure variations. As a result, we will focus on the crystal and amorphous ice, and also have an introduction to the pressure dependence of liquid water and SCW.

### 3.1. Different Forms of Ices

**Figure 4:**
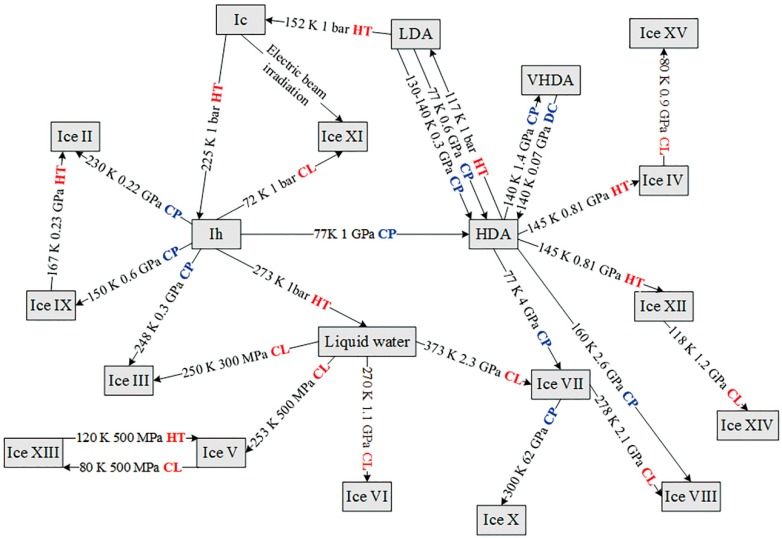
Schematic of 29 phase transition processes between different water phases. The temperature and pressure for the phase transitions are shown in the center of the arrows, and the 11 processes labeled with CP or DC in blue denoting as compression or decompression, respectively, are characterized by pressure dependence. While, the 17 processes labeled with HT or CL in red representing for heating or cooling, respectively, have the feature of temperature dependence.

There are sixteen or so kinds of crystalline ices, in which each water molecule obeys the ice rules. Namely, every water molecule bonds with four neighbors through HBs. Besides, three distinct amorphous ices, namely low density amorphous ice (LDA), high density amorphous ice (HDA) and very high density amorphous ice (VHDA), have also been found to occupy three obviously different megabasins in the energy landscape [89], and involve large amount of dangling OH bonds. For the three amorphous ices, they can be obtained under specific temperature and pressure conditions. Commonly, low temperature stimulates the ordered arrangement, and high pressure reduces the distances between neighbor molecules, as a result of which, temperature and pressure variation can induce water structure changes appearing as specific phase transitions. In Figure 4, there are 16 crystal ices and three amorphous ices involved in 29 phase transition processes, among which 11 processes are engendered by compression or decompression and 17 processes are induced by temperature variation.

Considering that two different species (e.g., ES and CS) coexist in liquid water, which is deduced in Section 2.2, this structural features would extend to the low temperature state, as the variation of water HB network lags behind the circumstance changes. It has been found that LDA was associated with ES as evidenced by the Raman scattering spectra [44], their almost identical density [90], and inelastic X-ray spectra [91]. Relative to LDA, VHDA has a dielectric relaxation time of two orders of magnitude shorter [92], which means the distorted HB network and the more freedom of orientational motion of individual water molecule. So, considering the structure relationship between supercooled water and LDA expounded in Section 2.1, it could be speculated that VHDA and HDA are related to CS. In addition to single phase, transition processes between amorphous phases are also the key to finding out the inheritance relationship and further picking up a suitable water model. As the ES has a density of 0.94 g·cm^−3^, and the density of its partially puckered form is 1.18 g·cm^−3^, which is remarkably similar to the density of HDA (*i.e.*, 1.17 g·cm^−3^). In case that it is not a coincidence, just like ES and CS, LDA and HDA can be viewed as two components in the LDA→HDA process. Excitingly, a series of intermediate forms [93], and even simultaneous presence of LDA and HDA [94], have been found in this phase transition process, which could serve as a strong evidence for continuous nature of phase transition. Based on this, we can presume that, LDA-HDA and LDL-HDL, are the concrete representations of ES-CS, and coexist in amorphous ice and liquid water, respectively.

The difficulty in breaking up the compiled snowman and the ease to recombine fractured ice stick together seem to be contradicted to the slippery feature of ice surface. Intuitively, when skating or skiing, the frictional heating promotes the generation of thin water film, which can facilitate the relative movement between the ice and other object, while, its refreezing is responsible for the underlying stickiness of ice. Nevertheless, another explanation on the occurrence of water film is the pressure melting effect [95]. However, as for intact ice ground, there is not enough time to get the surface ice melted to facilitate the relative movement, so the detailed mechanism is waiting for further experimental and theoretical verification. Another amazing effect caused by high pressure up to 1.76 TPa, is the transition of ice to the “metallic” water possessing free electrons that can move throughout the substance. This material, bearing high conductivity, can be used as the “metallic” conductor at some extreme conditions in the future.

### 3.2. Liquid and Supercritical Water

Identical to our intuition, pressurization of liquid water from ambient condition to 200 MPa, induces an increase in density which has been related to other anomalies, e.g., decrease in viscosity [96], increase in sound speed [97]. So, the anomalies in macroscopic parameters can be employed to conjecture the pressure-induced microscopic structure changes in liquid and supercritical water system.

**Figure 5:**
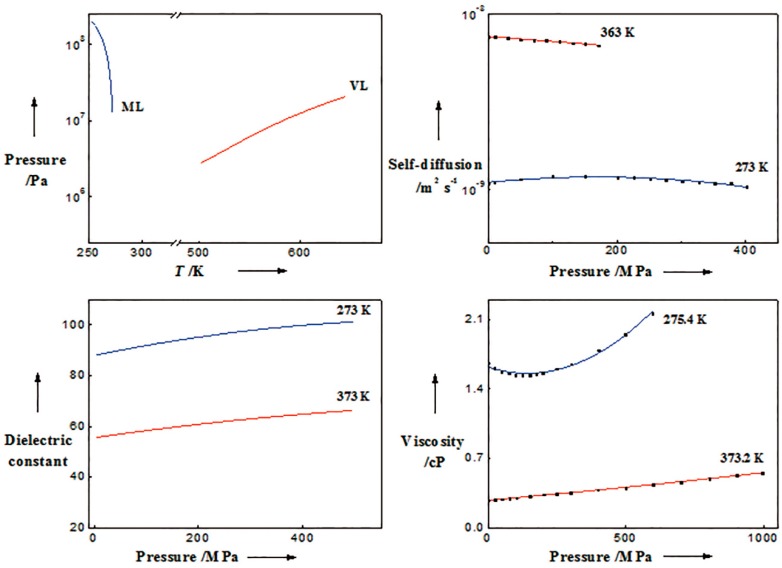
Pressure-dependent variations in four macroscopic parameters within different temperature ranges or at typical temperature points. There are two lines in each panel characterized by blue and red color representing for the low and high temperature lines, respectively. The left panel in the top line shows the melting line and vaporization line adapted from Dunaeva *et al.* [101], and Chaplin [102], respectively. The pressure dependence of self-diffusion, dielectric and viscosity are shown in other three panels which are adjusted from Prielmeier *et al.* [98], Uematsu and Frank [99], and Bett and Cappi [96], respectively.

From the phase diagram of water with pressure and temperature as vertical and horizontal axis, respectively, we could see the melting line is characterized by a negative slop, while the vaporization line is just on the contrary, which means the densification of water decreases ice’s melting point and increases liquid water’s boiling point. A deeper insight into the structural features of liquid water should be related to the pressure-dependent variations in macroscopic parameters under specific temperature. Upon incessant pressurization, the diffusivity of supercooled water was found to increase initially and then decrease, but the diffusivity of ambient water (e.g., at 363 K) descended constantly [98]. Moreover, the anomalous dielectric constant- [99] and viscosity-pressure behavior [96], can be viewed as the consequences of structural ordering and the balance between hydrogen bonding and van der Waals dispersion force. The variations in four parameters with pressure can be seen from Figure 5.

So it could be speculated that high pressure forces neighboring water molecules to interact with each other by HB from a more freedom state with intensive thermal motion, but the pressurization of water at low temperature (e.g., 273 K) does damage to the HB network that has already formed and promotes the arrangement of molecules in a more disordered manner, which is supported by the decline of viscosity with pressure ascending to 200 MPa (see Figure 5). This pressure-dependent variation features of structure are similar to the observations based on quantitatively matching between MD-DFT calculations and experimental findings [100].

Besides liquid water, SCW are also significantly pressure-dependent. Compression of water to high pressure at specific temperature induces dielectric constant elevation [103], and red shift of O–D symmetric stretch vibration of D_2_O in H_2_O [80], so HB in SCW would be strengthened upon compressing. In addition to pressure, density is another parameter used to describe physical state of SCW. Utilizing reverse Monte Carlo simulations, Jedlovszky [82] found the tetrahedral arrangement preferred in ambient water, would disappear with density decreasing from 0.890 to 0.724 g·cm^−3^ reflected by the probability distribution and the tetrahedral order parameter. However, there is some incompleteness of this theoretical method in considering the quantum, thermodynamic and geometric mechanisms. In this context, an experimental study, occupying X-ray diffraction and NMR, further manifested that SCW bore hydrogen bonds even at a low density of 0.7 × 10^−3^ g·cm^−3^, while the number of nearest neighbor water molecules decreases monotonously from 4.4 at ambient condition to 1.7 at the density of 0.7 × 10^−3^ g·cm^−3^ [104]. Hence, even under supercritical condition, water possesses a magic structural motif that will be discussed in the following part.

For amorphous ice, within the framework of ES-CS model, the inherent units composed of 14 water molecules [38], are confined within their local spaces by hydrogen bond and van der Walls forces exerted by the units nearby. So the only way to resist further compression is to reshape the perfectly tetrahedrally-coordinated units into distorted motif, reflected by the shortened dielectric relaxation time of VHDA. Nevertheless, for ambient water, the units loosely interact with each other, or even have enough interstitial spaces for them to rotate and translate. Consequently, the units can get close to each other without destroying their nature configuration upon compression, which leads to the formation of larger expanded structure. However, this model encounters a problem of explaining the long-range disorder of SCW, which may result from the oversize of cluster unit. Hence, as expounded in Section 2.3, choosing size-variable basic unit is an alternative way to show the long-range disordered arrangement of SCW manifested by neutron diffraction experiments combined with MC simulations [79].

Protein denaturation stems from not only temperature variation, but also pressurization. Under high pressure, the empty cavities of structured proteins are forced to fill with external water molecules [105], which reshapes the energy landscape to facilitate denaturation. While as for SCW, compression of water dissolving polar compound, for example, MgSO_4_ (aq), could result in demixing followed by crystallization. However, simultaneous cooling and decompression of water dissolving nonpolar gaseous products of SCWO reaction to room conditions, may separate the stream into a liquid aqueous phase and a gas phase [106]. That is to say, artful control of water pressure could be used in the extraction and separation of different substances.

## 4. Solutes-Dependent Effect on Water

Different from temperature and pressure, solute should be deemed as a semi-external factor that has an effect on water cluster. However, it is inevitable that water structure would be irreversibly influenced by adding solute molecule into pure water. As a result, we will focus on the changes in HB network induced by inorganic ions, proteins and carbohydrates by employing ES-CS model, so as to shed light on some state of art of questions pertinent to the rationalization of classifying solutes into chaotropes and kosmotropes, and the long-range effect of solutes on water structure.

### 4.1. Effect of Inorganic Ions

It is well known that freezing point depression, together with boiling point elevation, vapor pressure lowering and osmotic pressure, are the colligative properties of water, which are dependent on the concentration of solutes. Hence, the structure changes induced by ions can be speculated.

By measuring water activity, Koop *et al.* [107] observed the presence of solutes, independent of their nature, had an effect on ice nucleation that is very similar to the application of pressure. So the interstitial spaces sensitive to pressure may play the key role in variations in water activity. Similarly, the variations in ionization and compressibility with solute concentration was also related to the occupations of water clathrate cavities [102]. So one question comes into our mind. Could only volumetric effect of solute molecule or the reduction in molar fraction of water account for the deviations of macroscopic properties from ideal behavior? Unluckily, the Raoult’s law, neglecting the nature of solutes, fails to describe vapor pressure depression behavior for a series of solutes at high concentrations, for example, the concentrated CaCl_2_ solutions at 100 °C [108]. On the other hand, it is found that cations, e.g., Li^+^, are capable of gathering a specific number of water molecules to form primary hydration shell where the water molecules are considerably polarized compared to gas-phase structures [109], and anions, e.g., Cl^−^, may also create a specific connection mode between themselves and neighboring water molecules [110]. Hence, besides volumetric effect, it is essential to take other factors into consideration, e.g., interaction via electric fields of ions, polarization of water molecule, and put forward reasonable and universal classifications of different ions so as to reveal the mystery of ion-dependent water cluster changes.

**Figure 6:**
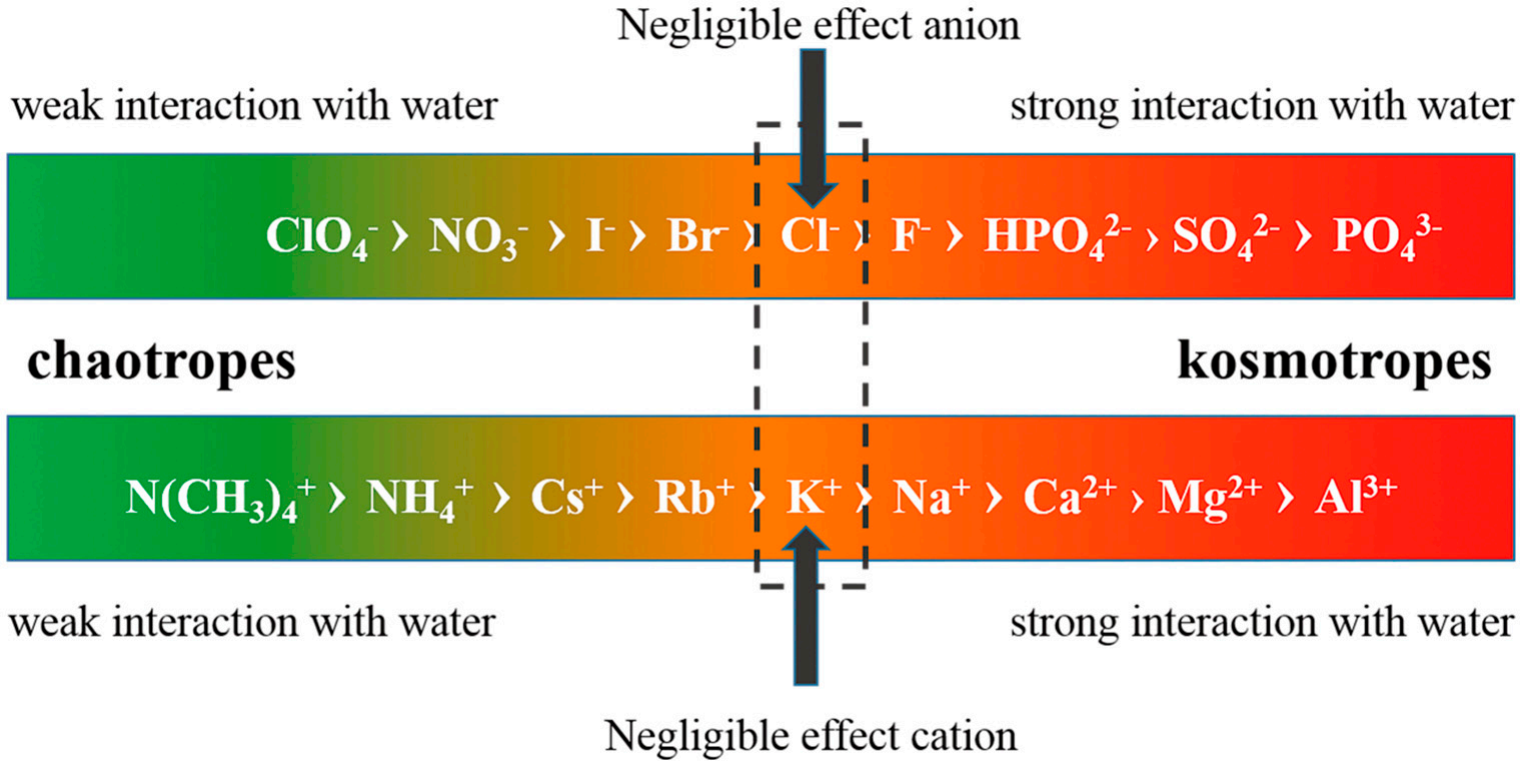
Schematic of relative intensity of interactions of nine anions and nine cations with surrounding water molecules, based on the definition of chaotropes and kosmotropes. The ions on the left side of the two sequences have weaker interactions with water relative to those on the right side.

In 1985, considering an ion’s ability in altering water structure, Collins and Washabaugh [111] came up with the concept of structure-breakers or chaotropes and structure-makers or kosmotropes. Consequently, this classification promoted tremendous studies on testing its applicability in distinct situations. Unluckily, researchers faced dilemma when explaining some phenomena by holding this criterion [112]. As mentioned above, the variations in properties under different conditions could be explained by the shift of local equilibrium within the framework of ES-CS model, so it is suitable to classify ions into kosmotropes and chaotropes according to their ability in shifting the equilibrium to more CS and more ES, respectively, by measuring the macro- and microscopic properties. Accordingly, anions and cations follow specific orders shown in Figure 6.

According to this classification, both anions and cations could be divided into kosmotropes and chaotropes depending on the extent of interactions of these ions with surrounding water molecules. In effect, Zavitsas [113], defining mole fraction (*x*) of solute particles as Equation (2), has observed the deviations of four colligative properties stemming from less solvent present than the conventionally calculated stoichiometric total by least square fitting the experimental data to the four calculation models.

(2) $$x=iM_{s}\;/\;(M_{w}-hM_{s}+iM_{s})$$

The term *hM*_s_ is the missing or strong bound water, as distinguished from “bulk” or “free” water. This model enables a magnificent extension of linear relationship between solute concentration and four properties, and evidences the formation of hydration shell. However, to give a more complete picture of hydration shell, the detection methods, capable of differentiating dynamical changes induced by ions, e.g., 2D IR spectroscopy, are good choices.

The dynamics of HB network is pretty fast in pure water. Specifically, the spectral diffusion time for HDO/D_2_O system and the vibrational lifetime in pure H_2_O, obtained from ultra-fast laser measurement, are less than 200 fs [114], and ~200 fs [115], respectively. Excitingly, Cowan *et al.* [116] broke through the hindrance of getting access to the fastest process of liquid water, and found the timescale of energy redistribution within the HB network was less than 50 fs by using 2D IR spectroscopy. Hence, we can define the influence of ions on the dynamics of hydration shell by comparing the two parameters of pure water to those of aqueous solutions. Kropman and Bakker [117] found halogenic anions could form weak HBs with water molecules in the first hydration shell, which further prolongs vibrational lifetime and spectral diffusion time to be 2.6–3.7 and 12–25 ps, respectively, being dozens of times longer than those in bulk liquid water. Recently, a 2D IR vibrational echo spectrum study also reported a slightly slowdown of water dynamics in NaBr solutions [118].

There is another interesting and counterintuitive case, which is correlated with ion-mediated water structure, *i.e.*, net chaotrope has the capability of increasing the viscosity of poly-electrolyte solution due mainly to the more low density water created between the polymers. On the contrary, in the absence of polymer, chaotrope alone has the opposite effect. And, as for the gas molecules evenly dissolved in the solution, the reduction in its solubility by adding strongly hydrated ions [119], could be tentatively explained by the long-range influencing mechanism. Therefore, spatial range that one ion exerts its power on is another question to be answered to define the influence of ion on the surroundings by using some site-specific detection techniques.

**Figure 7:**
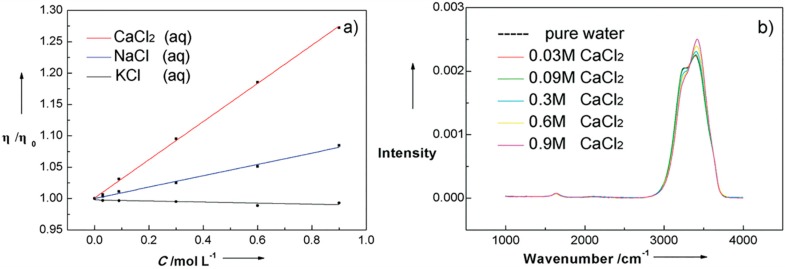
(**a**) Viscosity changes of three aqueous solutions with concentration. Among the three aqueous solutions, CaCl_2_ (aq) has stronger ability in elevating water viscosity; (**b**) Comparison of Raman stretching vibration spectrums of CaCl_2_ aqueous solutions with different concentrations. The Raman spectrum are normalized to have identical area from 2800 to 3800 cm^−1^ to facilitate the comparison.

The ion-dependent changes in water structure could be observed by spectrum study with varying ion concentration. An X-ray diffraction study observed the number of water molecules around KCl unit in aqueous solution was 45, with which clathrate like spherical structure formed [120], and based on topology, the KCl unit could influence more than three hydration layers. In 2012, Suresh *et al.* [121] used statistical mechanics to probe into the structure changes induced by cations and found the cation-induced changes in molecular orientation and non-linear polarization effects could persist up to ~3–4 hydration layers in the case of low charge density ions, and ~7–9 layers for high charge density ions. The same year, an IR photodissociation study on water nanodrops involving ions, manifested the ion-induced effect on water structure propagated all the way to the distance more than 1 nm from the ion, corresponding to 250 water molecules [122]. Not only so, the very long-range water structure can also be altered in a different manner relative to the hydration shell due to the cooperative effect. Very recently, a dielectric relaxation study on different aqueous solutions indicated the opposite effects of ions on water structure, namely, decelerating effect within the hydration shell and breaking effect beyond the hydration shell [123]. Coincidentally, by measuring viscosity and Raman spectrum of CaCl_2_ aqueous solutions, we observed viscosity increasing and absorption intensity decreasing between 3000 and 3300 cm^−1^ with CaCl_2_ concentration (see Figure 7). Interestingly, this phenomena could further be interpreted as the formation of robust hydration shell around Ca^2+^, while for water beyond this range, the HBs are loosened to discourage the formation of large- and middle-sized water cluster.

In view of this, ions can have a direct influence on water molecules within a couple of hydration layers, nevertheless, the interactions between water molecules far from ions changes a lot because of the cooperative effect among water molecules and electric fields exerted by ions. So it is imperative to show the long-range distribution features of water molecules in real space by using macro- and microscopic testing methods simultaneously in the future.

Hydration water has proved to be the root of functioning of proteins, e.g., facilitating energy transfer from hydration water to protein, influencing enzymatic reaction. Ions, as additives, can affect the stabilization of proteins by alternating water structure. Specifically, chaotropes prefer the ES-like low-density water cluster resemble to the water structure around protein’s hydrophobic groups, consequently, the isolation of protein molecules is enhanced with the presence of chaotropes. On the contrary, kosmotropes stimulate the precipitation (*i.e.*, salting out) by breaking ES-like water clusters between protein molecules. Besides protein, lower molecular weight hydrophobes, such as polyene antibiotics, can be also stabilized/destabilized by adding chaotropes/kosmotropes. On this basis, different kinds of separations, e.g., separations of enzymes and cell membranes, could be achieved by adding specific chaotropes or kosmotropes into solution systems [124].

### 4.2. Effects of Proteins

**Figure 8:**
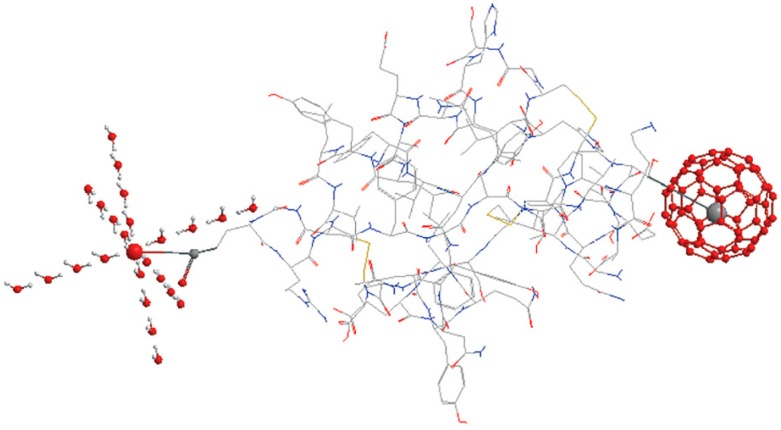
Schematic of two structural motifs of water cluster around polar and non-polar group of insulin molecule. Most of the insulin molecule is displayed in wire frame except for one carboxylate (above left) and one methyl (above right) shown in ball and stick mode. The magnified oxygen atom of carboxylate is surrounded by CS-like water cluster. The magnified methyl is surrounded by ES-like cluster of 60 water molecules.

As the key biomacromolecule for creatures, protein is composed of polar groups, e.g., carboxyl and amino, and non-polar groups, e.g., aliphatic chain. Both types of groups create ordered water structure around them, but the order created by them is extremely different (see Figure 8). For example, the carboxylate oxygen atoms can directly hydrogen bond to 4 [125] or 6 [126] water molecules, as a result, a localized high density water (CS-like structure) would form around the carboxylate groups, while for the methyl, a localized low density water (ES-like structure) would be created because of the strengthening of hydrogen bond between water molecules.

From the perspective of dynamics, hydration water is profoundly affected by the slow but large-scale motion of protein molecule, reflected by dielectric susceptibility and relaxation frequency of hydration water [127]. Meantime, two types of water-network relaxation behaviors with distinct energy and time distributions was observed for apomyoglobin [128]. If the hydrophobic surface of protein is taken as sphere, the distinct dynamics may be orientation dependent, *i.e.*, the OH bonds tangent to the surface and the dangling OH bonds pointing toward the surface. The former having stronger HB relative to the latter, even to that of bulk water, bear the feature of slower reorientational behavior [129]. That is to say, hydrogen bond switching events are greatly hindered by the extended hydrophobic surface of protein, which is further evidenced by a 2D IR study tactfully employing a metal carbonyl vibrational probe [130]. Recently, an MD study aided by bioinformatics analyses identified the perturbation of protein inter-helical hydrogen bonds can couple to rapid changes in water dynamics [131]. Consequently, besides the nature of bulk water, protein structure can also enslave the dynamics of hydration water.

In addition to the influence on vicinal water, functional groups of protein can also have a long-range effect on water structure supported by THz spectra, MD simulation, *etc*. In 2007, Ebbinghaus *et al.* [132] found the nonlinearity of THz absorption with protein concentration, which was attributed to the onset of overlapping of dynamical hydration layers, and concluded the hydration shell around protein extended to at least 9 Å, consistent with the femtosecond-resolved fluorescence spectrum study on dynamical hydration layer extending to more than 10 Å [128]. Furthermore, Ding *et al.* [133] estimated a hydration layer thickness of ~12–17 Å from peptide surface by using terahertz time domain spectroscopy (THz-TDS). Recently, on the basis of “hydration layer overlap” hypothesis, Bye *et al.* [134] used a modified calculation model (Equation (3)) to explain the variation in absorption coefficient with BSA concentration.

(3) $$\alpha_{\mathrm{total}}=\alpha_{\mathrm{bulk}}\left(\frac{V_{bulk}}{V_{tot}}\right)+\alpha_{shell}\left(\frac{V_{shell}^{tot}}{V_{tot}}\right)$$

Upon which, by assuming a cubic lattice distribution of protein molecules within the solution, the distance variation impact is shown in Equation (4).

(4) $$\frac{V_{shell}^{tot}}{V_{tot}}=\frac{V_{shell}}{l^{3}}\;=\;c\{\begin{array}{c} V_{shell}^{0}\;if\;c\;\leq \;{\left(2R\right)}^{-3}\\ V_{shell}^{0}\;-\;6V_{cap}\;if\;{\left(2R\right)}^{-3}\;<\;c\;\leq \;{\left(R\;+\;r\right)}^{-3} \end{array}$$

The key to calculating the effective distance affected by protein is *V*_cap_, which is determined by Equation (5).

(5) $$V_{\mathrm{cap}}=\frac{\pi h^{2}}{3}\left(3R-h\right)=\frac{\pi }{3}\left(2R^{3}-\frac{3}{2}\frac{R^{2}}{c^{1/3}}+\frac{1}{8c}\right)$$

Using this model, the authors further extended the hydration layer to be 15 Å from protein. However, this model does not take the absorption of BSA within the frequency range of 0.3–3.3 THz into consideration, which makes this model lose its universality. It should be noted that some studies, on the contrary, conclude the protein dominates only one hydration layer rather than multi-hydration layers [135,136]. The controversy may originate from the distinct definitions of hydration layer, sensitivity of apparatus, just like the case of ionic hydration layer. So it would be helpful to use time-resolved and site-specific detection methods to characterize the hindered relaxation process and long-range order (see Figure 8) in the future.

### 4.3. Effects of Carbohydrates

As typical carbohydrates, saccharide molecules, characterized by abundant hydroxyls with high electronegativity, could directly form many HBs with surrounding water molecules. In this part, we will try to illustrate the features of interaction between saccharide and water molecules.

As a result of the stronger ability of saccharide hydroxyl in forming hydrogen bonds with surrounding water molecules relative to that of water hydroxyl, it is intuitive to imagine a disrupted water cluster around saccharide molecule [137,138]. On this basis, a molecular simulations study concluded the steric constraints imposed by carbohydrate molecule was the reason for the disruption of tetrahedral arrangement [139]. Therefore, the number of water molecules in the first hydration layer serves as a good indicator of disturbing effects imposed by different carbohydrates. A small number for disaccharide (*i.e.*, trehalose), has been estimated to be 7.8 [138]. Meanwhile, it was reported the number of water instantaneously hydrogen bonding to carbohydrates, no matter for disaccharide or monosaccharide, was about 1.8 per OH [139], in other words, the value is ~14.4 for disaccharide. Recently, a Gigahertz-to-Terahertz Light Scattering and MD simulations study found a larger number, ~3.3 water molecules per hydroxyl to be part of the hydration shell of mono- and disaccharide [140]. So the hydroxyl plays the key role in determining the structure of water molecules nearby.

The dynamic differences between hydration and bulk water is another reflection of the impact of saccharide on surrounding water. Given the short HB lifetime of 0.9 ps for bulk water, a THz study accompanied by molecular simulations predicted a longer value of 1.3 ps for hydration shell to give a better fit to the HB correlation function [141]. Shortly thereafter, a more pronounced retardation of 1.9 ps was observed for the first solvation layer of trehalose by molecular simulations [142]. Hence, the dynamic of hydration water is not in their “native” state, but strongly affected by the presence of sugar molecule. However, what about the water molecules beyond the primary hydration layer? Put another way, does the hydroxyl have long-range effect on water structure?

It has been long believed that saccharide and surrounding water are responsible for the ability of creatures in protecting themselves from violent collision. So the key question is how large the hydration layer is to show the obvious bio-protection. Heugen *et al.* [141] assumed an averaged distinct absorption coefficient with a stepwise change from hydration water to bulk water and further estimated the hydration layer might extend to 5.13 ± 0.24 Å from lactose by fitting THz spectral data to the three-component equation (Equation (6)).

(6) $$\alpha {\left(\omega \right)}_{\mathrm{total}}=\frac{V_{solute}}{V}\alpha {\left(\omega \right)}_{solute}+\frac{V_{SW}}{V}\alpha {\left(\omega \right)}_{SW}+\left(1-\frac{V_{SW}}{V}-\frac{V_{solute}}{V}\right)\alpha {\left(\omega \right)}_{bulkwater}$$

Note that this model is almost identical to Equation (3) but one additional term incorporated to describe solute absorption. Further, Heyden *et al.* [142] proved the thickness of hydration shell is ~5.7–6.5 Å for disaccharides (*i.e.*, lactose and trehalose), and ~3.7 Å for glucose by occupying similar method. Very recently, by artificially decomposing THz time domain spectroscopic data, it was found that disaccharide was capable of controlling more water molecules as hydration water than monosaccharide [143]. Thus, sugar molecule as an ensemble has cumulative effect on surrounding water, which depends on the hydroxyl number.

**Figure 9:**
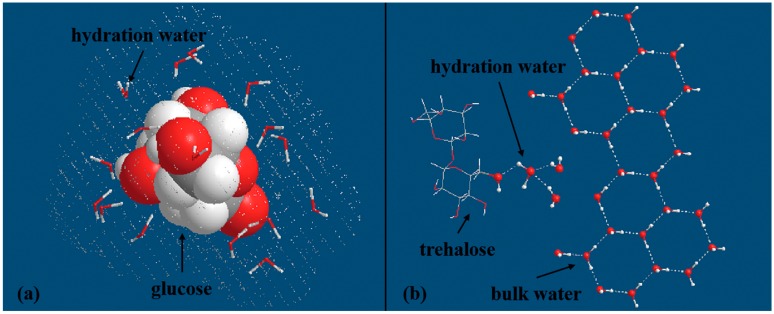
(**a**) Schematic of glucose and the first hydration layer consisting of 15 water molecules, as inferred from Lupi *et al.* [140]; (**b**) Schematic arrangement of many ordered bulk water molecules and two hydration layers controlled by trehalose, as inferred from Sajadi *et al.* [144].

In effect, saccharide molecules have close or even direct interactions with each other by excluding abundant surrounding water molecules without the occurrence of precipitation because of the abundant hydroxyls. So the model mentioned above, assuming cubic lattice distribution of sugar molecules, cannot be applied to the highly concentrated solutions. Coincidentally, a group using extended frequency range depolarized light scattering spectroscopy (EDLS), observed a pretty fast decreasing in hydration number with increasing sugar concentration, which was attributed to the occurrence of solute aggregation [140]. After all, EDLS has low time resolution, which makes it incompetent to detect the fast structure changes. So, the THz experiments with higher time resolution, aided by attaching a molecular probe on trehalose, could further improve the space resolution without sacrificing any time precision, and has confirmed the region influenced by disaccharide molecule to be larger than the first two solvation layers [144]. Even so, compared with protein and ion, saccharide has remarkable shorter-range effect which is the consequence of lower electronegativity and smaller number of hydroxyl relative to those of carboxylate and azyl. Consequently, saccharide may be unable to stimulate the formation of large-scale expanded structure. The impact on first hydration layer and the long-range effect induced by sugar molecule could be seen from Figure 9. Up to now, we are sure that the dynamics and number of hydration water in the first hydration layer are remarkably influenced by the presence of hydroxyl. However, the space controlled by sugar molecules are still debated, which necessitates further high level ab initial calculation studies to confirm the affected structural motif.

## 5. Mystery Effects Imposed by External Fields

Due to the delocalization of electrons held among water molecules, water can interact with external fields, *i.e.*, electric field, magnetic field and electromagnetic field, and further present some amazing phenomena, e.g., the water bridge [145]. Substantially, affected by external fields, the HB network is necessarily reshaped, which can be detected by various methods.

### 5.1. Effect of Electric and Electromagnetic Field

Water molecule has large dipole moment and strong polarizability, so it is intuitive to speculate the enhancement of HB strength and the line alignment of water molecules in the direction of external electric field. As a result, the self-diffusion coefficient of treated water is lowered down as compared with that of untreated water [146]. It seems that electric field can strengthen hydrogen bonding and increase the degree of association, however, maybe it is not just the case. About 20 years ago, an ^17^O-NMR study has confirmed the decrease of mean water cluster size with the presence of electric field [147]. Electric field has also been shown to remarkably alternate the phase transition of water in open system [148], which results from the partial or complete destruction of HB network. So, it is supposed to analyze the study results with comparable experiment conditions to tell apart the electric field-induced effect on water structure.

In effect, the influence on water cluster is dependent on the strength of external electric field. It is reported the freezing process would be inhibited with the presence of very strong electric field (5 × 10^9^ V·m^−1^) [149]. Nevertheless, the promoted ice formation under weak field (10^5^ V·m^−1^) [150], indicates the weakening of hydrogen bonding. Additionally, even for the electric fields with similar strength, the electrically treated water can also behave utterly different under complex conditions. For example, bread can get stiff because of water loss, but bread treated by electric field of 50 kV, would maintain softness longer, which is the result of the increased amount of free water molecules, or in other words, the raised water activity [151]. On the contrary, a research occupying axisymmetric drop shape analysis-electric fields (ADSA-EF), suggested the surface tension of water increased by about 2% when 7.5 kV electric field was applied [152]. So, another reason for the opposite effect on water may be the direction of HB relative to the external field. External electric field can facilitate the reorientation of HBs in the direction of the field and further the transmission of hydrogen ions [153], while the HBs orthogonal to the field are remarkably weakened at the meantime [154].

Electromagnetic radiation, generated by varying electric and magnetic field, is demonstrated to exert its power mainly through the electrical effect [155]. So, similar to the case of electric field alone, electromagnetic field can also reshape water structure in totally opposite manner under different conditions. As a result, there are some divergences on the effect of electromagnetic field on HB network. It has been shown the stretching vibration absorption of water was affected by weak electromagnetic field, concretely, the lower energy part of absorption spectrum was weakened, which was interpreted as the decreasing of coherent population accompanied by the increasing of intermediate population [156]. However, Shen, treating water with extremely low frequency electromagnetic field, found the dielectric constant of treated water was 3.7% higher than the control over the frequency range of 1–10 GHz [157], which indicates stronger polarization and higher degree of association in the treated water. It should be pointed out that the amplitude of field (0.15 T) in the former study is 3000 times larger than that (45 μT) in the latter study. Hence, the effects of electromagnetic field on HB are also strongly dependent on the strength of external field.

Electromagnetic radiation has been widely used in the microwave heating with industrial and domestic purpose. The oscillating electric field is able to force water dipole moments to reorient [155], the frictional interaction between reorienting molecule and unaffected or lag molecule leads to energy loss by heating. This process is largely dependent on the strength of HB; qualitatively, this effect could be invoked at GHz frequencies (microwaves) for ambient liquid water, whilst the frequency descends down to KHz (long electromagnetic waves) for solid ice. Additionally, electromagnetic field has biological effects as well. An experiment reported that electromagnetically conditioned water had both beneficial and adverse effects on the growth of higher organisms at optimum and higher levels of conditioning, respectively [158], so this effect can be applied in stimulating the growth of beneficial organisms or inhibiting the unwanted microorganisms.

### 5.2. Effect of Magnetic Field

Existing in nature, the magnetic field has also been observed to have an influence on water structure reflected by thermodynamic properties, spectrum, *etc*. An MC simulation of liquid water found significant changes in internal energy, heat capacity and further the radial distribution function with increasing magnetic field intensity [159]. Additionally, IR absorptions and Raman scattering spectrum of magnetized water, are also observably different from that of untreated water [160], and evidence the structure changes. However, there are completely opposite viewpoints held on the magnetic field-induced effect on water cluster.

The $n_{\mathrm{HB}}$ is a direct marker to show the changes in water structure imposed by magnetic field. When water is exposed to magnetic field, the weakening of hydrogen bonding and consequently the decrease of $n_{\mathrm{HB}}$ were observed by an MC simulation [159]. Given the noncoincidence between positive and negative charge center of water molecule, magnetic field can force water molecule to change its motion trail. So the weakening effect of magnetic field is due primarily to the presence of Lorentz force. Nevertheless, an IR study found the red-shift of near-infrared peak of water around 1900 nm under very intensive magnetic field of 14 T [161], subsequently, the refractive index of magnetized water under 10 T was observed to increase by ~0.1% [15]. In 2009, a comprehensive research found the higher activation energy and slower rotational dynamics of magnetized water, which was attributed to the increase in the average size of water clusters [162]. The strengthening of hydrogen bonding, evidenced by these observations, can be explained by the magnetic field-dependent balance between hydrogen bond and van der Waals force. The magnetic field does do damage to the van der Waals force as demonstrated by the controllable Zeeman predissociation process [163], as a result, the HB would be favored by the treated water.

In any case, magnetic field can exert its power on water structure. Precisely because of this, the applicability of NMR in detecting the changes in water cluster would be challenged. However, thanks to the ‘memory’ effect, namely the effect lasting for a period of time, other non-real time detection methods, e.g., IR stretching vibration absorption [164], can be employed to deduce the water structure changes. Unluckily, another problem is present here. The memory time is dependent on the field intensity and treating time, so it’s hard to keep all water samples measured under the same conditions and within the same time interval after treating, which makes the research findings less comparable between each other. So the next step is to develop integrated *in-situ* detection technology and to quantify the memory effect reasonably.

Even though the influencing mechanism has not been fully understood, the magnetic fields have been widely used in water-mediated processes, among which the magnetic field-assisted inhibition of scale formation appeals many attentions to do the mechanism research. One explanation is that the composition of crystalline polymorphs of CaCO_3_ is changed with the presence of magnetic field [165]. Meanwhile, a field study in power station gave an alternative explanation that the crystallization of carbonates was blocked due to the initiation of another competitive process, *i.e.*, the activation of colloidal silica by deforming the diffuse layer mainly consisting of water molecules [166]. So, no matter for which interpretation, the scale inhibiting effect is related to the deformation of water structure, as further evidenced by higher compressive strength of mortar samples mixed with magnetized water [167]. However, the HB network is strongly dependent on environment temperature, pressure and dissolved oxygen as expounded above, so fully understanding this mechanism necessitates broader studies on the influence of various factors on the treatment efficiency.

Meanwhile, magnetic fields have been found to have many biochemical and biological effects. For example, water interacting with solid surface weakly, responds to magnetic field and further bears more ordered structure around hydrophobic molecules [168], probably ES-like water cluster, which can prevent biomolecules from precipitating from solutions. Attractively, it was reported that blood viscosity could be reduced by applying magnetic fields of 1 T or above in the blood flow direction, and this effect was intuitionally ascribed to the formation of blood cell chains along the disk diameter direction [169]. As for blood with dispersed cells, there are abundant water molecules interacting with blood cells. While exposing blood to magnetic field, the changes in interstitial water cluster are still unknown and need to be characterized to define the role of water molecules in decreasing blood viscosity.

## 6. Summary and Outlook

A vast number of experimental and theoretical studies on water structure changes induced by four externalities, *i.e.*, temperature, pressure, solute and external field, are generalized, analyzed, and systematized in this work. It is out of doubt that the four factors could remarkably alternate the structure of water cluster. Therein, the temperature and pressure variation have the features of no introduction of any additional substance and good adjustability, with which vast results have been obtained to deduce the changes in HB network and water structural motif. Further considering the solute- and external field-induced structure changes, the modified ES-CS model is favored in explaining many water-related phenomena, even for supercritical water. In brief, the structural units are prone to tessellate to form expanded cluster with stronger HBs when water is exposed to specific externalities, e.g., low temperature and chaotropes. Broadening variation ranges of externalities, water cluster would gradually transit to collapsed structure with the structural unit shrinking in continuous mode, as exemplified by gaseous water.

Now, one can declare that ionic and non-ionic solute could exert their power on the first hydration layer with great confidence. Meanwhile, with the help of THz spectroscopy, MD simulations, *etc.*, these solutes seem to have a long-range effect on surrounding water molecules as well. Because of the differences in precision of detection devices and definition of hydration layer, the effective ranges affected by solutes are still remained to be resolved. Very recently, Guo *et al.* [170] presented a submolecular-resolution imaging of individual water monomers and tetramers to discriminate the orientation of monomers and the HB directionality of tetramers in real space. This technique is promising to get the space arrangement of several layers of water molecules visualized, so as to confirm the changes in water structure in the vicinity of various solutes.

In comparison to the other three externalities, there are fewer studies on the effect of external fields on water cluster. Even for the limited studies, the utterly opposite conclusions have been obtained for the same external field, which is caused by the *ex-situ* detection and the interference of instrument, e.g., NMR. Hence, *in-situ* detections are strongly recommended to account for this problem, which rely on the creation of sample cell made by magnetic shielding materials suitable for diverse spectroscopies.

In addition to the four expounded externalities, others, such as constrained space, special location, also have an influence on water cluster, which is manifested by the anomalous physicochemical properties of water. For example, the single-layer freestanding graphene, within which the carbon atoms interact with water molecules through van der Waals force, is found to have potential in desalination with high performance due mainly to the high water permeability [171]. The surface water, characterized by the loss of HBs, has higher reactivity relative to the bulk water. Thus, the confined and surface water necessitate further explorations to pave the way for the better applications in the future.
